# Critical Evaluation of a microRNA-Based Risk Classifier Predicting Cancer-Specific Survival in Renal Cell Carcinoma with Tumor Thrombus of the Inferior Vena Cava

**DOI:** 10.3390/cancers15071981

**Published:** 2023-03-26

**Authors:** Mischa J. Kotlyar, Markus Krebs, Antonio Giovanni Solimando, André Marquardt, Maximilian Burger, Hubert Kübler, Ralf Bargou, Susanne Kneitz, Wolfgang Otto, Johannes Breyer, Daniel C. Vergho, Burkhard Kneitz, Charis Kalogirou

**Affiliations:** 1 Department of Urology and Pediatric Urology, University Hospital Würzburg, 97080 Würzburg, Germany; 2 Comprehensive Cancer Center Mainfranken, University Hospital Würzburg, 97080 Würzburg, Germany; 3 Guido Baccelli Unit of Internal Medicine, Department of Precision and Regenerative Medicine and Ionian Area-(DiMePRe-J), School of Medicine, Aldo Moro University of Bari, 70124 Bari, Italy; 4 IRCCS Istituto Tumori “Giovanni Paolo II” of Bari, 70124 Bari, Italy; 5 Department of Pathology, Klinikum Stuttgart, 70174 Stuttgart, Germany; 6 Department of Urology, Caritas St. Josef, University of Regensburg Medical Center, 93053 Regensburg, Germany; 7 Physiological Chemistry I, Theodor-Boveri-Institute, Biocenter, University of Würzburg, 97074 Würzburg, Germany

**Keywords:** kidney cancer, RCC, venous infiltration, biomarker, miR, risk stratification

## Abstract

**Simple Summary:**

Renal cell carcinomas (RCCs) can build a so-called tumor thrombus and grow into the renal vein and the vena cava, thereby representing a high-risk situation for affected patients. Nevertheless, there are substantial differences in the clinical courses of RCC patients with a tumor thrombus. Currently, there is no established biomarker which helps identifying patients with a substantial risk of tumor relapse or even cancer-related death. Previously, members of our group discovered a signature of three small RNA molecules (specifically: microRNAs) in RCC tissue, which significantly predicted cancer-related survival. In this study, we validated this signature in a larger cohort of patients suffering from RCCs with a tumor thrombus. Notably, stratifying our patients according to this microRNA signature nearly separated our cohort into two halves, which significantly differed in terms of clinical risk. Our research could help identifying high-risk patients in need for additional therapy, while sparing others from unnecessary treatments.

**Abstract:**

(1) Background: Clear cell renal cell carcinoma extending into the inferior vena cava (ccRCC^IVC^) represents a clinical high-risk setting. However, there is substantial heterogeneity within this patient subgroup regarding survival outcomes. Previously, members of our group developed a microRNA(miR)-based risk classifier—containing miR-21-5p, miR-126-3p and miR-221-3p expression—which significantly predicted the cancer-specific survival (CSS) of ccRCC^IVC^ patients. (2) Methods: Examining a single-center cohort of tumor tissue from *n* = 56 patients with ccRCC^IVC^, we measured the expression levels of miR-21, miR-126, and miR-221 using qRT-PCR. The prognostic impact of clinicopathological parameters and miR expression were investigated via single-variable and multivariable Cox regression. Referring to the previously established risk classifier, we performed Kaplan–Meier analyses for single miR expression levels and the combined risk classifier. Cut-off values and weights within the risk classifier were taken from the previous study. (3) Results: miR-21 and miR-126 expression were significantly associated with lymphonodal status at the time of surgery, the development of metastasis during follow-up, and cancer-related death. In Kaplan–Meier analyses, miR-21 and miR-126 significantly impacted CSS in our cohort. Moreover, applying the miR-based risk classifier significantly stratified ccRCC^IVC^ according to CSS. (4) Conclusions: In our retrospective analysis, we successfully validated the miR-based risk classifier within an independent ccRCC^IVC^ cohort.

## 1. Introduction

In approximately 10% of all cases, clear cell renal cell carcinomas (ccRCCs) extend into the inferior vena cava (ccRCC^IVC^) [1,2,3]. Although constituting a high-risk setting in general, there still is substantial clinical heterogeneity within the ccRCC^IVC^ subgroup—with reported 5-year survival rates ranging from 37% to 65% for non-metastasized patients treated with nephrectomy in combination with tumor thrombectomy [4,5,6,7,8,9]. Regarding this discrepancy, biomarkers are urgently needed to identify patients with a specifically high risk of cancer relapse [10,11]. Potentially, ccRCC^IVC^ patients may also benefit from adjuvant systemic therapy and an intensified follow-up.

microRNAs (miRs) as biomarker candidates are post-transcriptional regulators of gene expression in various cancer entities [12,13,14]. Regarding ccRCC, several studies have demonstrated the prognostic impact of miR expression levels in tumor tissue [15,16,17]. Previously, Vergho et al. established a combined risk classifier for patients with ccRCC^IVC^ receiving nephrectomy and thrombectomy in curative intention [10]. Based on miR-21-5p, miR-126-3p, and miR-221-3p expression in tumor tissue, the risk classifier significantly stratified patients regarding cancer-specific survival (CSS) in a single-center cohort (*n* = 37)—with a 5-year CSS of 78% vs. 18% in the favorable subgroup compared with the unfavorable subgroup [10].

**Figure 1:**
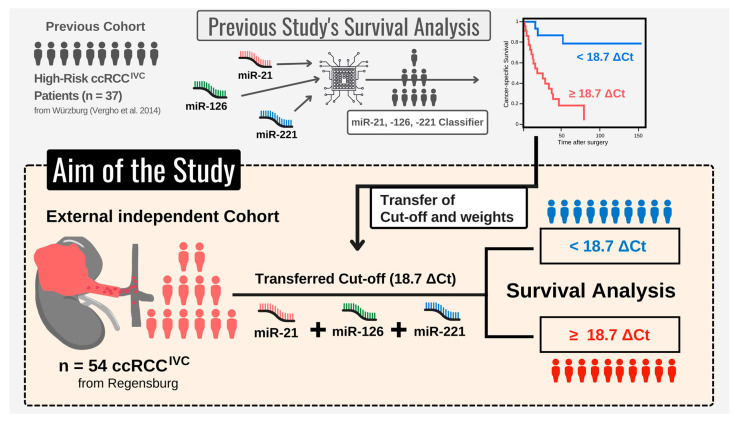
Course of the study—using a previously established microRNA (miR)-based risk classifier [10], we examined the prognostic impact of miR-21, miR-126, and miR-221 expression in an independent cohort of clear cell renal cell carcinoma samples with infiltration of the inferior vena cava (ccRCC^IVC^; *n* = 54). To assess the transferability of the miR-based risk classifier, cut-off values and weights were identical to the previous study.

To further assess the miR-based risk classifier as a prognostic tool in ccRCC^IVC^ patients, we retrospectively evaluated it within an independent cohort (*n* = 56) from the Department of Urology, University of Regensburg (Regensburg, Germany). Cut-off values for miR expression levels, as well as internal classifier weights, were transferred from the previous pilot study [10], in order to test its transferability to independent study cohorts. Figure 1 illustrates the course of our study.

## 2. Materials and Methods

### 2.1. Tumor Tissue Samples and Patients

Paraffin-embedded primary ccRCC^IVC^ tumor-samples of *n* = 56 subjects who underwent radical surgery were aggregated by the Department of Urology, University of Regensburg, Germany (1997–2006). A uropathologist selected sample regions with >90% cancerous tissue. Follow-up data were collected by the Department of Urology, University of Regensburg (Regensburg, Germany). The study was approved by the local Ethics Committee (Regensburg: Nr. 08/108). Detailed characteristics of the study cohort are summarized in Table 2.

### 2.2. RNA Extraction and qRT-PCR

Using the RecoverAll^TM^ Total Nucleic Acid Isolation Kit (Thermo Fisher Scientific, Waltham, MA, USA), total RNA from paraffin-embedded samples was isolated according to the manufacturer’s instructions. RNA concentrations and 260/280 ratios were analyzed by Spark^®^ 10M (TECAN, Männedorf, Switzerland). cDNA was synthesized from total RNA with stem–loop reverse transcription primers (TaqMan microRNA Assay protocol, Applied Biosystems, Birchwood, UK). The TaqMan microRNA Assay kit was used to quantify miR expression according to manufacturer’s protocols. Table 1 lists the specific primers and kits used in this study.

Samples exhibiting a standard deviation > 0.5 were excluded (all reactions performed in triplicates). Small nuclear RNA (RNU6B) expression was used for the normalization of relative miR expression values. Samples with expression levels of RNU6B > 30 Ct were excluded from further analyses. Relative miR expressions were calculated using the ∆Ct-method (∆Ct sample = Ct miR of interest − Ct RNU6B). To calculate fold changes in miR expression between samples, we used the 2∆∆Ct method (hereafter referred to as the ∆∆Ct method).

### 2.3. Statistics and Computational Analysis

A Jupyter Notebook environment (version 6.3.0) was used to perform all statistical analyses using Python version 3.8.8, LifeLines version 0.27.0 [18], Pandas version 1.2.4 [19], Matplotlib 3.3.4 [20], and SciPy version 1.6.2 [21]. To analyze differences between miR expression levels, we used Student’s *t*-tests for normally distributed data with similar variance—otherwise, the Mann–Whitney U test was applied. Data distribution and variance were assessed via Shapiro–Wilk and Levene tests, respectively. A significance level of 0.05 was applied. miR expression levels (reads per million mapped reads) from pT3b tumor samples belonging to the ccRCC cohort of The Cancer Genome Atlas (TCGA) were downloaded from the GDC portal (https://portal.gdc.cancer.gov, accessed on 12 March 2023). 

#### 2.3.1. Initial microRNA-Based Risk Classifier Calculation

The risk classifier from the pilot study was calculated as follows (for this initial study, different R/Bioconductor packages were used):

- Performing single-variable and multivariable Cox regression analysis, Vergho et al. evaluated the impact of clinicopathological parameters and various miRs on CSS.
- To select the best fitting Cox model, the relative goodness-of-fit was measured based on the Akaike information criterion (AIC) for different variable combinations. The combination of miR-21, -126, and -221 displayed the best prediction properties.
- Finally, the miR-based risk classifier was calculated based on the publication by Lossos et al. [22]. Hereby, the z-factor calculated by the multivariable Cox model (R package ‘survival’) for miR-21, -126, and -221 was multiplied with the relative expression levels (ΔCt) of the respective miR. This approach resulted in the following formula: (4.592 × ΔCt miR-21) + (−3.892 × ΔCt miR-126) + (−1.938 × ΔCt miR-221).
- Subsequently, a receiver operating characteristic (ROC) curve was plotted (R package pROC), showing the true positive rate (TPR) against the false positive rate (FPR) at various threshold settings. The risk score cut-off/threshold of 18.7 ΔCt was determined from ROC using the Youden’s J statistics [23] (integrated in pROC package [24], default settings).

#### 2.3.2. Clinical Validation of microRNA-Based Risk Classifier

miR-specific weights for individual ΔCt values and the cut-off value (≥18.7 ΔCt = “unfavorable subgroup”, <18.7 ΔCt = “favorable subgroup”) were transferred from the pilot study [10] and applied in order to stratify the ccRCC^IVC^ study cohort from Regensburg and perform Kaplan–Meier analyses. Within the risk classifier formula, a negative factor indicated that higher expression levels correlated with longer survival, whereas a positive factor correlated with shorter survival. For further analysis, we also transferred cut-off values for miR-21 (8.17 ΔCt), miR-126 (3.57 ΔCt), and miR-221 expression (1.84 ΔCt) to evaluate their predictive potential in the new cohort using the Kaplan–Meier survival analysis.

## 3. Results

Table 2 summarizes the basic clinical and pathological characteristics of our study cohort. Follow-up information was available for 56 patients, the precise date of death for two patients was unknown.

**Figure 2:**
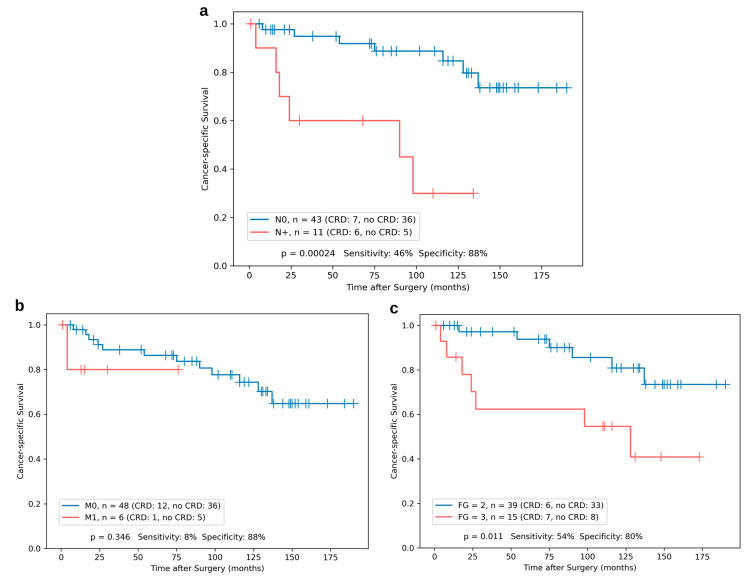
Kaplan–Meier survival analysis for cancer-specific survival for ccRCC^IVC^ (*n* = 54) cohort from Regensburg stratified by nodal status (N0 vs. N+, (**a**)), synchronous metastasis (M0 vs. M1, (**b**)) and Fuhrman grade (FG = 2 vs. FG = 3, (**c**)). *p* values from log-rank tests as well as sensitivity and specificity are shown within each plot.

To further characterize our patient cohort, we performed Kaplan–Meier analyses regarding the clinicopathologic parameters nodal status, synchronous metastasis and Fuhrman grade. As illustrated in Figure 2a, nodal status significantly stratified the patients regarding CSS (*p* = 0.00024). For synchronous metastasis (Figure 2b), stratification was not statistically significant. In contrast, CSS according to Fuhrman grade 2 vs. 3 (Figure 2c) was significantly different (*p* = 0.011). While being statistically significant for nodal status and Fuhrman grade, stratification based on these parameters only reached sensitivities of 46% and 54%, respectively. 

### 3.1. Association of miR-21, -126, and -221 Expression with Clinicopathological Characteristics

**Figure 3:**
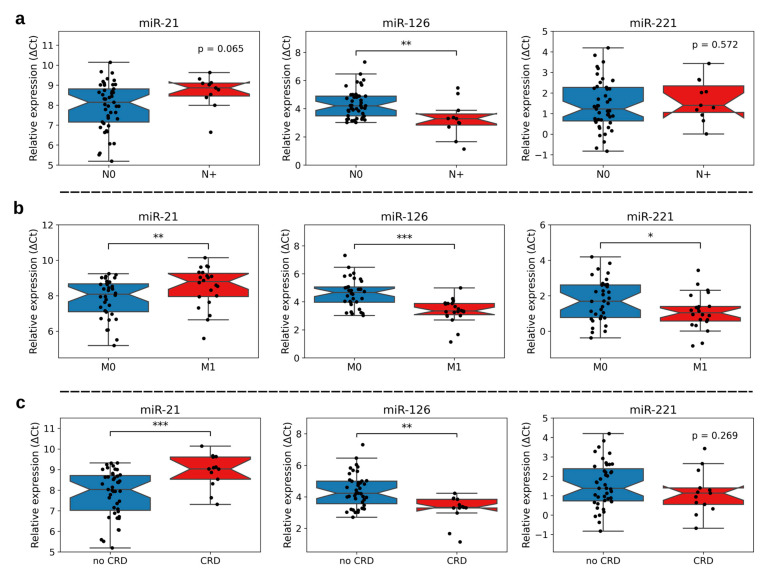
miR-21, -126, and -221 expression levels depending on lymphonodal status (**a**), distant metastases (synchronous and metachronous, (**b**)), and cancer-related death (CRD, (**c**)). Significant changes between subgroups were calculated using an unpaired Student’s *t* test (CRD: miR-221; nodal status: miR-21, -221; distant metastases: miR-21, -126) or Mann–Whitney U test (CRD: miR-21, -126; nodal status: miR-126; distant metastases: miR-221). * *p* < 0.05; ** *p* < 0.01; *** *p* < 0.001.

To investigate the impact of miR-21, -126, and -221 within our ccRCC^IVC^ cohort, we associated expression levels of miR-21, miR-126, and miR-221 with the relevant clinical parameters. Figure 3 illustrates the results.

At time of surgery, 11 of 56 ccRCC^IVC^ patients (19.6%) were diagnosed with lymphonodal metastasis. In cases with a positive nodal status, trends towards the up-regulation of miR-21 (*p* = 0.065) and a significant down-regulation of miR-126 (*p* < 0.01) were observed. For miR-221, there was no statistically significant association to nodal status.

Distant metastasis (synchronous and metachronous) emerged in 22 of 56 ccRCC^IVC^ patients (39.3%). As shown in Figure 3b, we observed a significant up-regulation of miR-21 (*p* < 0.01) and down-regulation of miR-126 (*p* < 0.001) as well as miR-221 (*p* < 0.05) in ccRCC^IVC^ samples of patients with metastasized disease. 

Of 56 patients with ccRCC^IVC^, 13 (23.2%) died during the follow-up period due to cancer (cancer-related death, CRD). Regarding miR expression levels, we found a significant up-regulation of miR-21 (*p* < 0.001) and a down-regulation of miR-126 (*p* < 0.01) in CRD cases. Instead, miR-221 expression did not show a statistically significant association with CRD in this analysis (*p* = 0.27).

For comparison, we also investigated the expression levels of miR-21, miR-126, and miR-221 depending on survival within the ccRCC cohort of the TCGA database (Figure S1). In line with our findings, the upregulation of miR-21 was significantly associated with death in patients with pT3b tumors. In contrast, miR-126 was significantly downregulated in these cases, whereas miR-221 levels did not differ significantly.

### 3.2. Single-Variable and Multivariable Cox Regression Analysis

Next, we performed single-variable Cox regression analysis to further assess the prognostic potential of miR-21, -126, and -221 expression levels as predictors of CRD. Detailed follow-up information was available for 54 of 56 cases, with a median of 94 months. 

As summarized in Table 3a, miR-21 and miR-126 significantly predicted the occurrence of CRD in our study cohort (*p* = 0.003, hazard ratio (HR) 3.79 for miR-21, *p* = 0.00003, HR 0.19 for miR-126). In contrast, miR-221 expression in tumor tissue did not display significant prognostic potential (*p* = 0.22). Regarding further clinical parameters, significant results were also observed for nodal involvement, metastatic status, and Fuhrman grade.

**Figure 4:**
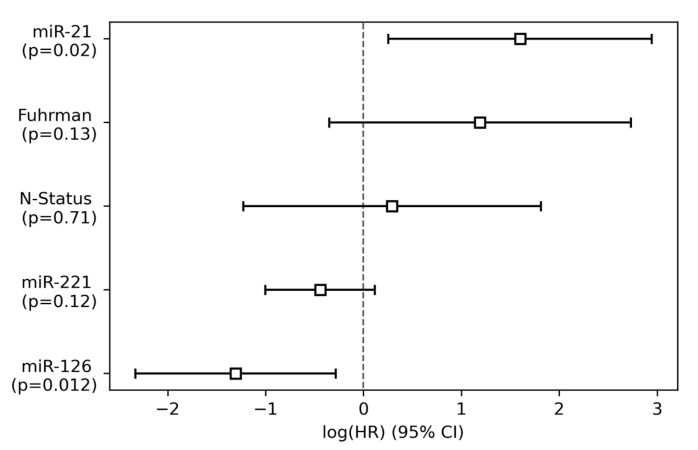
Forest plot representing log hazard ratios (HRs) from multivariable Cox regression of miR-21, miR-126, miR-221, Fuhrman grade (Fuhrman), and nodal status (N-Status) for cancer-related death (CRD). *p* values were computed using chi-squared tests.

Additionally, as shown in Table 3b and Figure 4, we performed multivariable Cox regression to investigate whether the miR candidates remained as relevant predictors of CRD in attendance of nodal involvement and Fuhrman grade. Again, miR-21 and miR-126 significantly predicted the occurrence of CRD (*p* = 0.02, HR 4.94 for miR-21, *p* = 0.01, HR 0.27 for miR-126). miR-221 expression again did not meet statistical significance as a predictor of CRD (*p* = 0.12) (Table 3 and Figure 4). No significant results were observed for the clinical parameters nodal status (*p* = 0.71) and Fuhrman grade (*p* = 0.13), either.

To further understand and illustrate the prognostic impact of single miRNAs on CSS (based on previously fitted multivariable Cox regression model), we plotted survival curves according to isolated miR expression levels (Figure S2). For miR-21, higher relative expression levels were associated with lower CSS (Figure S2a). In contrast, higher expression levels of miR-126, as well as miR-221, were associated with higher CSS (Figure S2b,c).

### 3.3. Kaplan–Meier Analyses for Single miR Expression and the Risk Classifier

**Figure 5:**
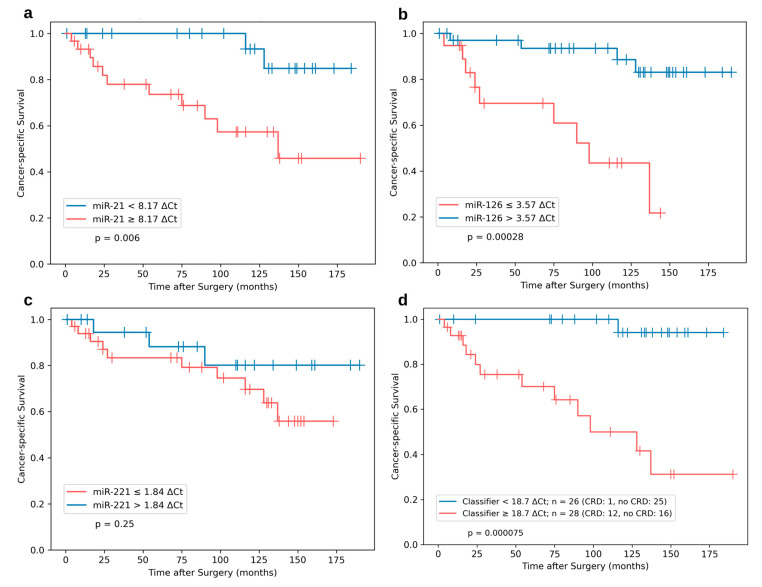
Kaplan–Meier survival analysis for CSS for the external independent ccRCC^IVC^ (*n* = 54) cohort from Regensburg stratified by miR-21 (**a**), miR-126 (**b**), and miR-221 (**c**) expression levels. (**d**) Combined miR-based risk classifier (miR-21, -126, -221) using identical cut-offs and weights from a previous publication [10]. *p* values from log-rank tests are shown within each plot.

To investigate the prognostic validity of single miRNA expression levels, survival analyses using the identical cut-offs from Vergho et al. [10] were performed. Both miR-21 (*p* = 0.006) and miR-126 (*p* = 0.00028) showed a strong predictive significance in the Kaplan–Meier survival analysis (Figure 5a,b). However, differences regarding CSS of miR-221 high vs. low expressing tumor specimens (Figure 5c) did not reach statistical significance (*p* = 0.25). As illustrated in Figure 5d, combing all three miRs within the risk classifier and maintaining the established cut-off level of 18.7 ΔCt (≥18.7 ΔCt = unfavorable subgroup, <18.7 ΔCt = favorable subgroup) revealed the most significant stratification (*p* = 0.000075). 

In conclusion, the risk classifier reached a sensitivity of 92.3% (CI 95%: 62.1–99.6%) and a specificity of 61.0% (CI 95%: 44.5–75.4%). Differences in 5-year and 10-year CSS were 100% vs. 70% and 94% vs. 31% between the favorable and the unfavorable subgroups, respectively.

### 3.4. Testing the Composition and Robustness of the Risk Classifier

In line with the results from the Würzburg cohort investigated in a previous publication [10], patient stratification based on single miR-221 expression did not demonstrate significant results. Therefore, we checked whether adding miR-221 expression to our Cox regression model contributed to its performance. Measuring the goodness-of-fit with the Akaike information criterion (AIC), the combination of miR-21, miR-126, and miR-221 again emerged as the best fitting Cox model with the lowest partial AIC (Table S1). The coefficient for distant metastases (Table 3) was not estimable within Cox regression (positively infinite); therefore, we did not include this variable in our analysis.

Finally, we investigated the robustness of the risk classifier in terms of the varying cut-off values applied. We calculated sensitivities, specificities, and *p* values for cut-offs ranging from 16.0 ΔCt to 25.0 ΔCt (Table S2). In general, all cut-offs produced highly significant results. Higher values (21.0 ΔCt to 25.0 ΔCt) revealed a patient stratification with even lower *p* values; this rise in significance was accompanied by a decline in sensitivity—from 92% to 85% for the cut-off values 21.0–24.0 ΔCt and 62% for the cut-off value 25.0 ΔCt. Kaplan–Meier curves for the cut-off values 16.0, 17.0, 20.0, and 22.0 ΔCt are shown in Figure S3.

## 4. Discussion

ccRCCs infiltrating the inferior vena cava represent a clinically relevant high-risk subgroup. However, there was substantial clinical heterogeneity within this distinct subgroup—biomarkers were needed to assess the individual risk of progression. To address this, researchers have investigated the influence of clinical characteristics—showing the prognostic impact of variables such as perinephric fat invasion, body mass index, metastasis, and Fuhrman grade [8,25,26]. Regarding our in-house cohort, synchronous metastasis did not meet statistical significance—probably due to small sample size and limited follow-up. Although nodal status and Fuhrman grade demonstrated statistical significance, low sensitivities severely limited clinical applicability in our patient cohort. 

In line with nomograms and prediction models in metastasized RCC [27,28], researchers have also investigated the prognostic role of blood counts in RCC cases with tumor thrombus. Specifically, serum gamma-glutamyltransferase (GGT) [29], as well as neutrophil-to-lymphocyte ratio [30,31] and lymphocyte-to-monocyte ratio [32], were reported to significantly predict survival in patients suffering from RCC with tumor thrombus.

In general, adjuvant therapy with tyrosine kinase inhibitors (TKIs) or immune checkpoint blockers [33] could be a promising therapeutic option after nephrectomy—especially for patients suffering from high-risk RCC. However, European kidney cancer guidelines currently do not contain strong recommendations towards adjuvant therapies due to the mixed outcome in clinical trials [34]. For the TKI sunitinib, one trial indicated improved disease-free survival (DFS) for patients—although showing no significant differences in overall survival (OS) [35]. Additionally, another phase 3 trial did not identify significant survival effects for adjuvant sunitinib or sorafenib in nonmetastatic high-risk renal cell carcinoma [36]. Due to the sobering TKI results, research efforts have mainly shifted towards immune checkpoint blockers. For the PD-1 (Programmed Cell Death Protein 1) inhibitor pembrolizumab, the KEYNOTE-564 trial identified improved progression-free survival (PFS) in an adjuvant setting after nephrectomy [37].

### 4.1. Evaluating an miR-Based Risk Classifier for RCC with Infiltration of the Vena Cava

To estimate the individual risk of patients suffering from ccRCC^IVC^, members of our research group have established a risk classifier based on the tissue expression of miR-21, miR-126, and miR-221 [10]. The former cohort contained tumor tissue of *n* = 37 patients undergoing surgery at the University Hospital of Würzburg, Germany. In this study, we externally validated the prognostic potential of the miR-based risk classifier. Therefore, we examined an independent cohort of ccRCC^IVC^ from the University of Regensburg, Germany (*n* = 56). To test the transferability and usability of the classifier within an external tissue cohort, we applied identical cut-off values and weights as in the previous pilot study.

In terms of clinicopathological characteristics, miR-21 expression was significantly higher in patients with a positive lymphonodal status, metastatic disease, and in cases of CRD. In contrast, miR-126 expression levels were significantly lower for these clinical scenarios. Within the single-variable Cox regression, both miRs also demonstrated prognostic significance regarding cancer-specific survival (CSS). Lower levels of miR-221 in tumor tissue and its association with CRD did not reach statistical significance. Beyond miR expression, Fuhrman grade, lymphonodal status, and the occurrence of metastasis emerged as prognostically relevant. Next, we added multivariable Cox regression for the three miR candidates. Again, this study identified miR-21 and miR-126 expression levels to significantly predict CRD. Kaplan–Meier analyses based on cut-off values previously determined by Vergho et al. [10] confirmed the significant role of miR-21 as well as miR-126 expression levels for CSS prediction. Finally, the miR-based classifier was applied, using identical cut-off values and weights to split patients into two groups. 

Notably, the combined risk classifier nearly stratified the study cohort into two halves—with *n* = 26 patients belonging to the favorable subgroup and *n* = 28 patients belonging to the unfavorable subgroup. Regarding the substantial difference in CSS between both groups, adjuvant therapies appear promising especially for the unfavorable subgroup of ccRCC^IVC^ patients.

Given the fact that miR-221 failed to demonstrate statistical significance within single-variable and multivariable analysis, we re-checked whether combining the expression levels of all three miRs represented the ideal constitution of the risk classifier. As previously demonstrated [10], AIC analysis again confirmed that taking into account miR-221 expression improved the overall performance of the Cox regression model. 

We finally challenged the robustness of the applied cut-off value (18.7 ΔCt) by examining the results of patient stratification in terms of sensitivity, specificity, and significance. Except for the neighboring cut-off 19.0 ΔCt, higher levels of significance were only reached with lower sensitivities. In conclusion, the risk classifier demonstrated its transferability not only regarding its components, but also in terms of the previously established cut-off value. 

### 4.2. Functional Roles of miR-21, miR-126, and miR-221 in Cancer and Thrombosis

After confirming the prognostic potential of the miR classifier using the validation cohort from Regensburg, we were interested in the previously reported functions of these miRs in RCC and other malignancies. For miR-21, several researchers have demonstrated oncogenic effects in various cancers, including RCC [38,39]. Among the prominent miR-21 target genes are key players of apoptosis induction such as PDCD4 (Programmed Cell Death 4) [40] and genes such as PTEN (Phosphatase and Tensin Homolog) [38]. The latter is an established tumor-suppressor gene best known for regulating PI3K/Akt signaling. In contrast to miR-21, miR-126 acts as a tumor suppressor in tumor tissue, e.g., by targeting ROCK1 (Rho-Associated Coiled-Coil Containing Protein Kinase 1) [41] and VEGFA (Vascular Endothelial Growth Factor A) [42]. For miR-221, oncogenic versus protective functions appear to depend on the underlying cancer entity; researchers have demonstrated both roles [43,44,45]. For RCC, a downregulation of miR-221 appears well in line with previous publications. Specifically, miR-221 is reported to regulate KDR (Kinase Insert Domain Receptor)—also known as VEGFR2 (Vascular Endothelial Growth Factor Receptor 2)—in ccRCC [46] and prostate cancer [47], thereby regulating the sensitivity towards sunitinib. In summary, among diverse tumorigenic functions of these miRs, all three candidates prominently influence angiogenesis-related pathways (so-called AngiomiRs) [48,49]. Given that not all ccRCCs depend on angiogenic signaling to the same degree [50], it is tempting to assume that the unfavorable subgroup identified by our risk classifier could benefit from adjuvant anti-angiogenic therapy.

Notably, expression levels of these three miRs have reportedly been deregulated in thrombotic and embolic events [51]. miR-221 plasma levels, for example, were elevated in patients suffering from pulmonary embolism [52]. Moreover, Wang et al. found miR-21 to be part of an miR signature significantly associated with recurrent thromboembolism [53]. However, further research is needed to elucidate the potential impact of these miRs on a tumor thrombus. 

### 4.3. Limitations and Future Directions

Our study has several limitations. Leaving aside the definite RCC subgroup investigated here, sample size of our study is relatively small. Moreover, we purposely did not adjust cut-off values and individual miR weights determined previously in order to check the transferability of the risk classifier to external tissue cohorts. We additionally did not consider the operative duration, which could affect miR expression due to differing manifestations of tissue hypoxia. However, hypoxia does not solely depend on the length of the surgical procedure; it also depends on the surgical technique performed. Moreover, miR expression levels in primary tumors do not necessarily represent expression levels within cancer cells contained in the tumor thrombus. Warsow et al. investigated this potential discrepancy by performing whole-exome sequencing and found substantial heterogeneity regarding mutational signatures [54]. 

More research—ideally in a prospective setting—could further validate the risk classifier in a clinical setup and elucidate whether the sub-classification of ccRCC^IVC^ is able to identify patients most susceptible towards adjuvant therapy.

## 5. Conclusions

Although RCC extending into the inferior vena cava represents a high-risk setting, there is still substantial clinical heterogeneity within this patient subgroup. Previously, Vergho et al. established an miR-based risk classifier—containing miR-21, miR-126, and miR-221 expression—which significantly predicted CSS for patients from this subgroup. To validate this classifier, we examined its impact on an external and independent patient cohort. Using identical cut-off values for single miRs and identical weights within the classifier, we confirmed a highly significant risk stratification within the new cohort. Patients with an unfavorable constellation according to the miR-based classifier could especially benefit from adjuvant therapy and continuous follow-up examinations.

## Figures and Tables

**Table 1:** Overview of primers and kits used in the current study.

microRNA	Assay-ID	Catalog Number	Manufacturer
hsa-miR-21-5p	000397	4427975	Applied Biosystems
hsa-miR-126-3p	002228	4427975	Applied Biosystems
hsa-miR-221-3p	000524	4427975	Applied Biosystems
RNU6B	001093	4427975	Applied Biosystems
TaqMan™ Universal PCR Master Mix, no AmpErase™ UNG		4324020	Applied Biosystems
TaqMan™ MicroRNA Reverse Transcription Kit		4366596	Applied Biosystems

**Table 2:** Clinical and pathological patient characteristics (*n* = 56). Precise date of death for two patients was unknown.

Characteristics	
Median Follow-up (*n* = 54)	94 (1–190) months
Median Age at surgery	67 (41–89) years
Sex Female Male	22 (39.3%) 34 (60.7%)
Tumor Stage: pT3b	56 (100%)
Fuhrman Grade G2 G3	41 (73.2%) 15 (26.8%)
Nodal Status N0 N+	45 (80.4%) 11 (19.6%)
Distant Metastasis (synchronous and metachronous) M0 M1 (synchronous: *n* = 7, metachronous: *n* = 15)	34 (60.7%) 22 (39.3%)
Median Tumor Size	70 (18–225) mm
Overall survival yes no	27 (48.2%) 29 (51.8%)
Cancer-related death yes no	13 (23.2%) 43 (76.8%)

**Table 3:** (a) Single-variable Cox regression of ccRCC^IVC^ patients for miR expression levels and clinicopathological parameters; (b) multivariable Cox regression for miR expression levels as well as nodal status and Fuhrman grade. The 95% confidence intervals (CIs) are shown for hazard ratios (HRs). *p* values were computed using the chi-squared test. * *p* < 0.05; ** *p* < 0.01; *** *p* < 0.001.

	Cancer-Related Death			
	(a) Single-Variable Analysis		(b) Multivariable Analysis	
Parameters	HR (95% CI)	*p* Value	HR (95% CI)	*p* Value
miR-21	3.79 (1.55–9.26)	0.003 **	4.94 (1.29–18.98)	0.02 *
miR-126	0.19 (0.09–0.42)	0.00003 ***	0.27 (0.097–0.75)	0.01 *
miR-221	0.74 (0.46–1.19)	0.22	0.64 (0.37–1.12)	0.12
Age at surgery	0.98 (0.92–1.03)	0.42		
Sex	2.10 (0.58–7.65)	0.26		
Tumor size	1.01 (1.00–1.03)	0.07		
Fuhrman grade	3.79 (1.27–11.33)	0.02 *	3.28 (0.70–15.32)	0.13
Nodal status	6.70 (2.09–21.47)	0.001 **	1.34 (0.29–6.12)	0.71
Distant metastasis (synchronous and metachronous)	∞	NA		

Abbreviations: CI, confidence interval; HR, hazard ratio; ∞/NA: in case of distant metastasis as a predictor of CRD, the coefficient was not estimable (positively infinite). HR and *p* values are therefore not shown.

## Data Availability

The data presented in this study are available within the article or supplementary material.

## Supplementary Materials

The following supporting information can be downloaded at: https://www.mdpi.com/article/10.3390/cancers15071981/s1. Figure S1: miR-21 (a), miR-126 (b) and miR-221 (c) expression levels depending on overall survival status in pT3b ccRCC samples (*n* = 54) of The Cancer Genome Atlas (TCGA) database; Figure S2: Survival curves for varying miR expression levels of miR-21 (a), miR-126 (b) and miR-221 (c)—based on fitted multivariable Cox regression model; Figure S3: (a–d) Kaplan Meier survival analysis for CSS for external independent ccRCC^IVC^ (*n* = 54) cohort from Regensburg stratified by Combined miR-based risk classifier (miR-21, -126, -221) using various cut-off values.; Table S1: Summary of partial AIC (Akaike information criterion) values for different multivariable Cox model combinations; Table S2: Sensitivities, specificities and associated *p* values for the combined risk classifier with differing cut-off levels.
