# Supplementary material for: Critical Evaluation of a microRNA-Based Risk Classifier Predicting Cancer-Specific Survival in Renal Cell Carcinoma with Tumor Thrombus of the Inferior Vena Cava
Source: Cancers (Basel). 2023 Mar 26;15(7):1981. doi: 10.3390/cancers15071981 (PMC10093292; doi:10.3390/cancers15071981)
Supplement: Supplementary file 1 [file cancers-15-01981-s001.zip › cancers-2241851-supplementary.pdf]

Supplementary Materials

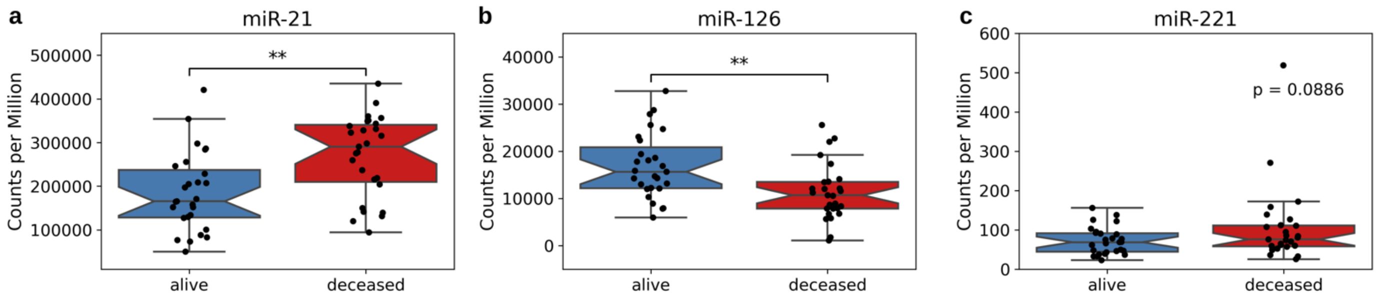

Figure S1. miR-21 (a), miR-126 (b) and miR-221 (c) expression levels depending on overall survival status in pT3b ccRCC samples (*n* = 54) of The Cancer Genome Atlas (TCGA) database. Significant changes between subgroups were calculated using unpaired Student's t test (miR-21, miR-126) or Mann-Whitney-U test (miR-221). $p < 0.01$ **.

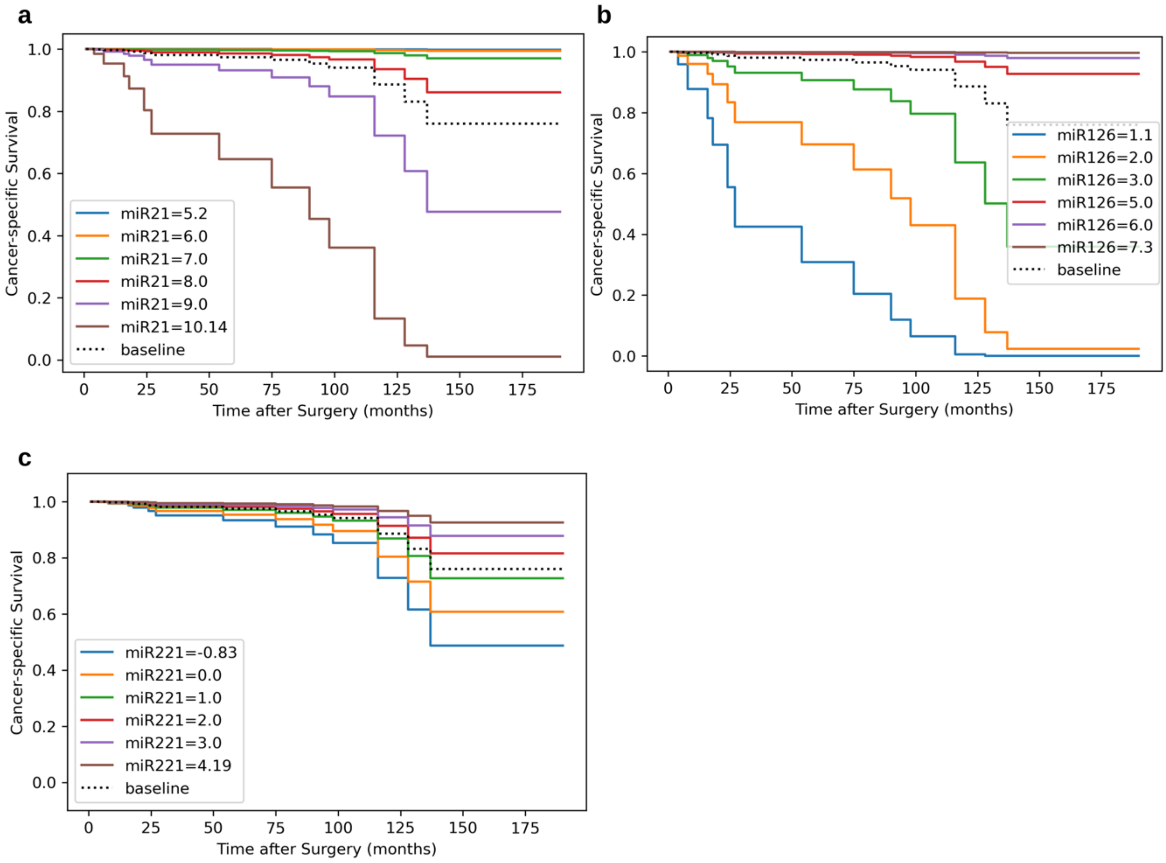

Figure S2. Survival curves for varying miR expression levels of miR-21 (a), miR-126 (b) and miR-221 (c) – based on fitted multivariable Cox regression model – illustrating partial correlations of single miRNAs on cancer-specific survival for present study cohort (*n* = 54). The baseline represents median relative expression for each miRNA (miR-21 = 8.34 ΔCt, miR-126 = 4.0 ΔCt, miR-221 = 1.3 ΔCt). Blue line represents minimum and brown line the maximum relative expression of each miRNA.

Table S1. Summary of partial AIC (Akaike information criterion) values for different multivariable Cox model combinations.

**Cox Model Combination**	**Partial AIC**
miR-21, miR-126, miR-221	60.67
miR-21, miR-126	61.14
miR-21, miR-126, Fuhrman Grade	61.16
miR-21, miR-126, Tumor Size	61.67
miR-21, miR-126, miR-221, Fuhrman Grade, Nodal Status	62.0
miR-21, miR-126, Nodal Status	62.76

Table S2. Sensitivities, specificities and associated *p* values for the combined risk classifier with differing cut-off levels.

**Cut-off** [ΔCt]	**Sensitivity [%]**	**Specificity [%]**	***p* value**
16.0	100	42	0.001009
17.0	100	51	0.000158
18.0	92	61	0.000075
18.7	92	61	0.000075
19.0	92	63	0.000053
20.0	85	71	0.000078
21.0	85	78	0.000005
22.0	85	85	2.29×10^{-7}
23.0	85	88	6.93×10^{-9}
24.0	85	88	6.93×10^{-9}
25.0	62	90	3.75×10^{-8}

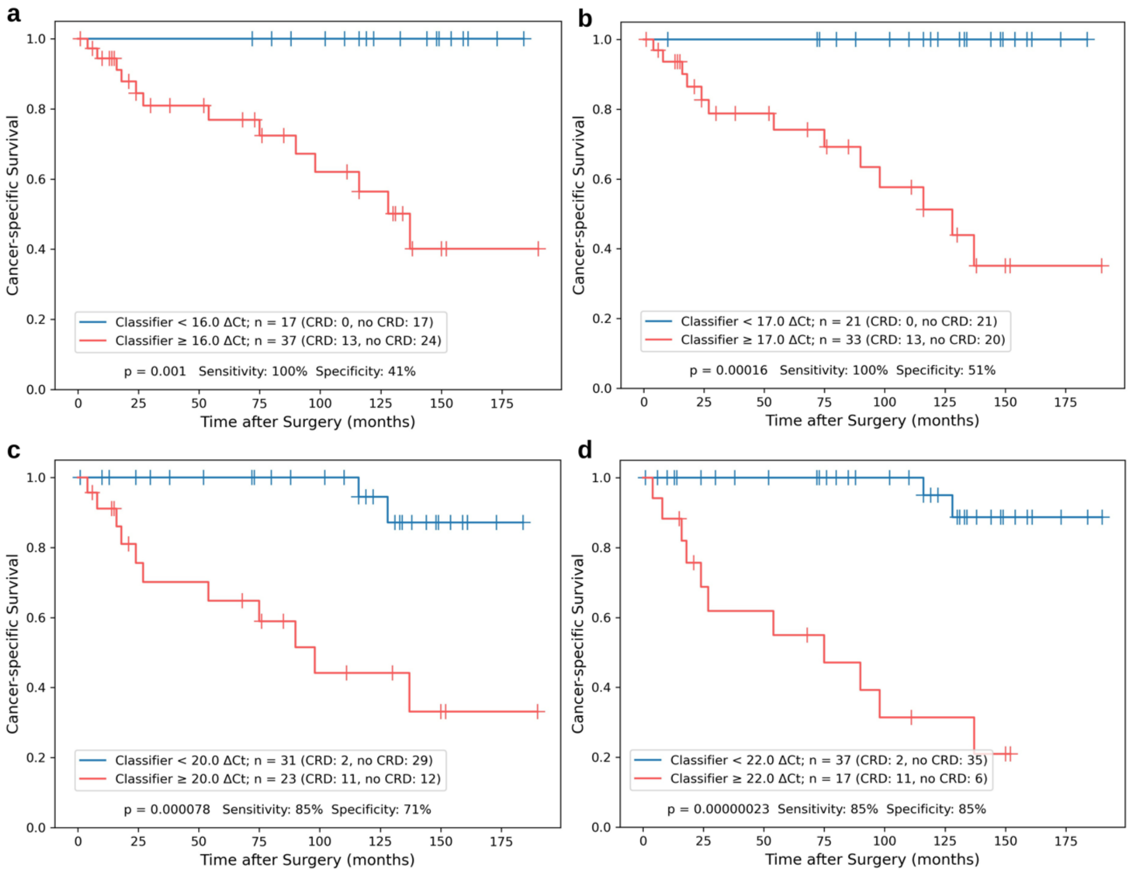

Figure S3. (a-d) Kaplan Meier survival analysis for CSS for external independent ccRCC^{IVC} (n = 54) cohort from Regensburg stratified by Combined miR-based risk classifier (miR-21, -126, -221) using various cut-off values. p values from log-rank tests are shown within each plot.
